# Multifaceted determinants of social-emotional problems in preschool children in Sweden: An ecological systems theory approach

**DOI:** 10.1016/j.ssmph.2023.101345

**Published:** 2023-01-21

**Authors:** Masoud Vaezghasemi, Thomas Vogt, Marie Lindkvist, Anni-Maria Pulkki-Brännström, Linda Richter Sundberg, Lisbeth Lundahl, Sven-Arne Silfverdal, Inna Feldman, Anneli Ivarsson

**Affiliations:** a Department of Epidemiology and Global Health, Umeå University, 901 87, Umeå, Sweden; b Department of Applied Educational Science, Umeå University, 901 87, Umeå, Sweden; c Department of Clinical Science, Pediatrics, Umeå University, 901 87, Umeå, Sweden; d Department of Public Health and Caring Sciences, Uppsala University, 751 22, Uppsala, Sweden

**Keywords:** Social-emotional health, Mental health, Preschool children, Ecological systems theory, Sweden

## Abstract

Social-emotional problems occurring early in life can place children at future risk of adverse health, social and economic outcomes. Determinants of social-emotional problems are multi-layered and originate from different contexts surrounding children, though few studies consider them simultaneously. We adopted a holistic approach by using Bronfenbrenner's process-person-context-time model as a structuring device. We aimed to assess what characteristics of families and children from pregnancy, over birth, and up to 3 years of age are associated with social-emotional problems in boys and girls. This study used regional data from the Salut Programme, a universal health promotion programme implemented in Antenatal and Child Health Care, and data from national Swedish registers. The study population included 6033 3-year-olds and their parents during the period 2010–2018. Distinct logistic regression models for boys and girls were used to assess associations between the family social context, parents' lifestyle, parent's mental health, children's birth characteristics, and indicators of proximal processes (the independent variables); and children's social-emotional problems as measured by the parent-completed Ages and Stages Questionnaire: Social-Emotional between 33 and 41 months of age (the outcome). Overall, a less favourable family social context, detrimental lifestyle of the parents during pregnancy, and parents' mental illness from pregnancy onwards were associated with higher odds of social-emotional problems in 3-year-olds. Higher screentime and infrequent shared book-reading were associated with higher odds of social-emotional problems. The multifaceted determinants of children's social-emotional problems imply that many diverse targets for intervention exist. Additionally, this study suggests that Bronfenbrenner's process-person-context-time theoretical framework could be relevant for public health research and policy.

## Highlights

• We use Bronfenbrenner's process-person-context-time model as a structuring device.
• Factors associated with 3-year-olds social-emotional problems are multifaceted.
• They include the family social context, and parents' lifestyle and mental health.
• Some indicators of proximal processes are associated with social-emotional health.
• The process-person-context-time model may be useful in public mental health policy.

## 1 Introduction

Children's social-emotional health relates to their experience and management of emotions, to learning processes, and to the establishment of meaningful relationships. Difficulties arising in young children's social-emotional health can have a detrimental impact throughout life: children with social-emotional problems are at greater risk of being stigmatized and of developing depressive symptoms through childhood and adolescence (Kaushik et al., 2016; Meagher et al., 2009). They may also be at greater risk of negative psychosocial-related outcomes such as substance use, mental health issues in adulthood, and involvement in crime (Fergusson et al., 2005; Jones et al., 2015); suicide (Caspi et al., 1996); and mortality into adulthood and at least up to middle age (Jokela et al., 2009). Social-emotional problems during childhood may also lead to worse educational and employment outcome in early adulthood (Jones et al., 2015).

There is ample evidence that the socio-economic circumstances in which children grow up impact their overall health, and their social-emotional health and development in particular (Pillas et al., 2014; Reiss, 2013). Parents' lifestyle such as maternal alcohol use (Easey et al., 2019; Lai et al., 2020), certain type of drug use (Lee et al., 2020), or smoking during pregnancy (Dolan et al., 2016; Lai et al., 2020), may also contribute to the onset of social-emotional problems in children. The parents own mental health problems can negatively impact the mental health of their children as well (Goodman et al., 2011; Lai et al., 2020; Weissman et al., 2006). Documented gender differences in social-emotional health suggest that young boys are at a higher risk of vulnerability than girls (Eurenius et al., 2019; Vaezghasemi et al., 2020). In addition, being born with an extremely low birthweight (Olsen et al., 2022) or preterm (Cheong et al., 2017; McIntosh et al., 2021) may have adverse consequences on social-emotional development. However, positive parenting can have a beneficial effect on children's social-emotional health (Mendelsohn et al., 2018).

From an equity perspective, it is concerning that inequalities originating at birth or before could persist over the life course (Pearce et al., 2020), possibly in part through early childhood social-emotional problems. Therefore, identifying and intervening on determinants of social-emotional problems in the early years could improve health, social, and economic outcomes during life, and contribute to reducing inequalities.

### 1.1 Bronfenbrenner's ecological systems theory and the process-person-context-time model

Bronfenbrenner's process-person-context-time model provides a theoretical framework for investigating potential determinants of children's social-emotional health in a holistic manner. It incorporates considerations on how forces that are both distal and proximal to the individual can influence its development. Although this framework has rarely been used in public mental health research and its usefulness is not well established for this specific research area, it has been proposed to have the potential to advance the field (Eriksson et al., 2018).

Composed of the elements of process, person, context, and time, this theory emphasizes the importance of proximal processes (“process”), which are the interactions occurring regularly over time between the developing child and other persons, and with objects and symbols. Proximal processes are seen as “the primary engines of development” (Bronfenbrenner, 2005a). Parent-child shared book-reading is an example of a proximal process that has been found to be a predictor of vocabulary development in very early childhood (Farrant & Zubrick, 2011). Similarly, parent-child interactions have been found to be associated with positive social behavior in children (Ashiabi & O'Neal, 2015). When it comes to interactions with objects and symbols, screentime has been found to be negatively associated with subsequent child development (Madigan et al., 2019).

Personal characteristics (“person”) relate to the biological, physical, physiological, or psychological characteristics of the developing child such as biological sex or birthweight. The effect of personal characteristics on a developmental outcome can be of interest, such as the impact of being born extremely preterm on social-emotional health (Olsen et al., 2022). However, personal characteristics may also moderate the impact of proximal processes and of more distal forces (Bronfenbrenner, 2005a; Bronfenbrenner, 2005b): the effects of proximal processes or contextual factors may for instance differ between boys and girls.

The context, refers to “the external world” and can be divided into four systems: the micro, meso, exo and macro systems. The microsystem is the environment in which the person develops, and where proximal processes take place. For young children, this could be the home or childcare. The mesosystem is made up of several microsystems, and of their interconnections. The exosystem is similar to the mesosystem in that it envelops several smaller systems and their interconnections, but is different in that it includes at least one environment that is not immediate to the child, such as the mother's workplace, or parents' contact with the healthcare system (Bronfenbrenner, 2005b). The fourth system is the macrosystem and relates to the overall configuration of a society. The type of labour market or the education system can be defining features of a macrosystem. Examples of the role of context in shaping child development include the effect of community socio-economic status or of maternal education on vocabulary development (Farrant & Zubrick, 2011).

Time can be understood as comprised of three levels. Microtime is concerned with the occurrence and continuity of a given proximal process (Bronfenbrenner & Morris, 1998). One could for example observe proximal processes including preschoolers as they happen in their home or in childcare (Tudge et al., 2003). Mesotime still relates to proximal processes, but is concerned with their frequency over longer periods, such as weeks (Bronfenbrenner & Morris, 1998). The number of times that adolescents meet with friends outside of school or work in a week is an example of such a lens on a proximal process (Benson & Buehler, 2012). The third dimension, macrotime, refers to evolutions over time in the society or beyond (Bronfenbrenner & Morris, 1998). Secular trends in the diagnosis of psychiatric disorders in children (Collishaw, 2015) are an example of this perspective.

### 1.2 Relevance of the current study

Holistic approaches to multi-layered determinants of children's social-emotional health could allow to identify targets for future research that could be translated into interventions, and Bronfenbrenner's theory provides such an approach. Sweden's free-of-charge health controls of 0–5 year olds, covering almost all children, provides an excellent case for making the kind of holistic analysis that the study is aiming at. In Västerbotten in northern Sweden, the Salut Programme has been raising healthcare workers and parents' awareness about children's social-emotional health in Antenatal and Child Health Care and previous work in this region has found that patterns of social-emotional problems among 3-year-olds differ based on gender (Eurenius et al., 2019; Vaezghasemi et al., 2020) and custody arrangement (Eurenius et al., 2019). Social-emotional health also appears to be socially patterned (unpublished results), with children growing up in unfavourable socio-economic circumstances being at higher risk of social-emotional problems. At this stage, knowledge coming from a holistic approach is needed to guide policies and interventions for preventing social-emotional problems before 3 years of age, and to promote social-emotional health at the population level.

### 1.3 Aim

Based on a holistic approach, this study aims to assess associations of families' and children's characteristics from pregnancy, over birth, and up to 3 years of age with social-emotional problems in boys and girls.

## 2 Methods

### 2.1 Study setting and data sources

This study relied on data collected as part of the Salut Child Health Promotion Programme, an ongoing intervention integrated into routine Antenatal and Child Health Care in Region Västerbotten in northern Sweden. Child Health Care has almost 100% coverage, similarly to preschools. As part of the Salut Programme, questionnaires were filled out by expecting parents before the first visit to Antenatal Care early in pregnancy, around gestational week 11. One health questionnaire was destined to the mother-to-be, and another one to her partner. Questions pertained to occupation, family situation, living conditions, and lifestyle. When the child turned 3, the parents were invited to fill out a questionnaire prior to an ordinary visit to Child Health Care. This questionnaire included the Ages and Stages Questionnaires: Social-Emotional (ASQ:SE) for 3-year-olds, for which the age range is 33–41 months-old (Squires et al., 2003), and which has been found to adequately identify children with social-emotional problems in this age range in Västerbotten (Vaezghasemi et al., 2020). The questionnaire also incorporated questions about the health, lifestyle and living conditions of the child. Data from the Salut Programme questionnaires were complemented with additional individual-level data from the National Board of Health and Welfare (Medical Birth Register and the National Patient Register) and from Statistics Sweden.

### 2.2 Study population

The study population (N = 6033) was a subset of the total records obtained from the 3-years-old questionnaires of the Salut Programme from 2014 to 2018, for children born from 2011 to 2015. During this time, 14,359 live births were recorded in Västerbotten (Statistics Sweden, 2022) and 10,689 3-years-old questionnaires were filled out as part of routine care. Approximately 75% of all the children living in the region thus have a questionnaire filled out for them by at least one of their parents or guardians. Questionnaires with no or missing parental consent for use of the data for research and those without a correct personal identity number were excluded. The remaining 9523 records were linked to the parents' questionnaires by Statistics Sweden. If a child questionnaire had no link to at least one parent's questionnaire, or if the personal identity number was detected as incorrect by Statistics Sweden, the record was excluded, and after that, 7680 records remained. Further exclusions were made based on mismatch between data from the parents' questionnaires (filled out during the pregnancy period) and the 3-years-old questionnaires, according to the following rules: if the date of birth of the child was more than 3 months away from the expected delivery date, the questionnaires of the mother and 3-year-old were not linked. Similarly, if the partner's questionnaire was filled out outside of a −8/+8 months period around the child date of birth, the questionnaires of the partner and 3-year-old were not linked. 6255 3-years-old questionnaires could be adequately linked to at least one parent questionnaire.

**Fig. 1:**
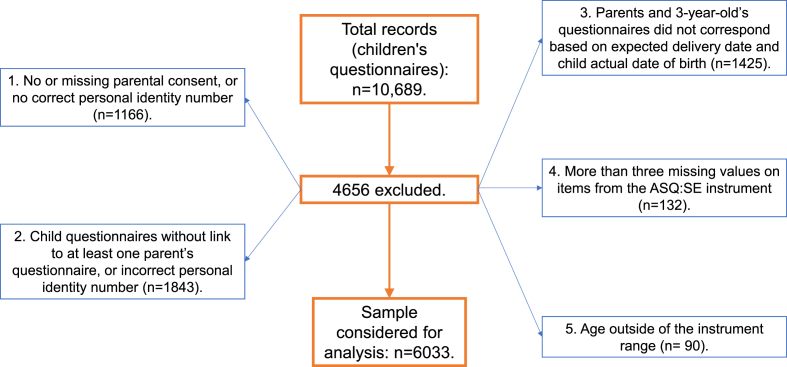
Flowchart describing the study population and the availability of the data.

Additionally, 132 children were excluded because their questionnaire had more than three missing values on questions from the ASQ:SE instrument (Squires et al., 2003). In addition, 90 had an age outside of the approved instrument range. The 6033 children included in the study corresponded to 56.4% of the initial records (Fig. 1). However, additional children were excluded from the final analysis if they had one or more missing values on independent variables, resulting in a sample size that varied based on the variables included in analyses.

### 2.3 Measures

#### 2.3.1 Outcome: social-emotional problems

Social-emotional problems were assessed by the ASQ:SE instrument from the 3-years-old questionnaire. A total score was obtained from 31 questions where the parents indicated how frequently a specific behaviour was occurring for their child, and if this was a concern for them. ASQ:SE scores can range from 0 to 465, and the cut-off point of ≥50 was used to identify children at risk of social-emotional problems, as it has been indicated as appropriate for a Swedish context (Vaezghasemi et al., 2022). To assess the robustness of the results to changes in cut-off point, and to allow for international comparisons, a second analysis was conducted using ≥59 as the cut-off point, which is the recommended cut-off value that is used internationally and is based on the US population (Squires et al., 2003). If a child had up to three missing values on the ASQ:SE items, the average value of the other items was used to replace the missing entries (Squires et al., 2003).

#### 2.3.2 Independent variables

Bronfenbrenner's process-person-context-time model was used as a structuring device to identify groups of variables that were conceptually related to each other. They are described below.

##### 2.3.2.1 Family social context (Context)

Parent's place of birth was obtained from Statistics Sweden and divided into three categories: (i) both born in Sweden; (ii) one born in Sweden; (iii) both born outside Sweden.

Family income was calculated from Statistics Sweden data as the mean of the inflation-adjusted disposable income of both parents for the three years preceding the calendar year in which the 3-years-old questionnaire was filled out. It was divided into quintiles.

Parental education was also obtained from Statistics Sweden and categorized as: (i) both parents have more than high-school education; (ii) one has more than high-school education; (ii) neither has more than high-school education. In 55 cases, information on educational attainment was available for only one parent and the parental education was then derived from that one parent. If the parent had more than high-school education, parental education was coded as “one parent has more than high-school education”. If the parent was high-school educated or less, parental education was coded as “neither has more than high-school education”.

Living arrangement was derived from the 3-years-old questionnaire and dichotomized as “both parents living together”: (i) yes; and (ii) no.

##### 2.3.2.2 Parents’ lifestyle (Context)

Variables relating to the parents’ lifestyle were self-reported early in pregnancy in the parents' questionnaires. Alcohol-related habits were assessed through the Alcohol Use Disorders Identification Test (Babor et al., 2001) for each parent individually, and then grouped and categorized into (i) neither parent with at-risk use or addiction; and (ii) one or both parents with at-risk use or addiction. Similarly, smoking habits were dichotomized into: (i) neither parent smokes; and (ii) one or both parents smoke. Drug use was divided as follows: (i) neither parent ever used drugs; (ii) one of the parents used drugs once or more; (iii) both parents used drugs once or more. Parental stress was classified as (i) neither parent feels stressed a lot; and (ii) one or both parents feel stressed a lot.

Whenever information was only available for one parent, the common variable for both was derived from that parent. As example for a binary variable, if smoking information was only available for the mother and she was not a smoker, the smoking variable was coded as “neither parent smokes”. If she was a smoker, the variable was coded as “one or both parents smoke”. For drug use, which had three categories, if information was available for only one parent and she/he never used drugs, the family variable would have been coded as “neither parent ever used drugs”. In the case where that parent had used drugs, the family variable would have been coded as “one of the parents used drugs once or more”.

##### 2.3.2.3 Parents’ mental illness (Context)

Information on parents' mental illness was obtained from the National Patient Register, which includes specialised outpatient visits and inpatient care episodes. Information about main diagnosis within the NordDRG system for classification of each healthcare encounter was used. Specifically, the Major Diagnostic Category 19, named “Mental illness, behavioral disorders and alcohol or drug addiction”, was used as the indicator for each parent's mental illness. Maternal and partner mental illness were operationalized as binary variables coded as “mental illness”: (i) yes; and (ii) no. Diagnostics were included from the calendar year preceding the child's year of birth and up to - and including - the year when the 3-years-old questionnaire was filled out.

##### 2.3.2.4 Personal characteristics (Person)

Sex was a binary variable obtained from Statistics Sweden and categorizing children as either girls or boys. Gestational age was obtained from the Medical Birth Register and categorized as (i) preterm; and (ii) normal. Preterm children were those born before 37 completed gestational weeks (World Health Organization, 2018). A third variable, birthweight, was also obtained from the Medical Birth Register and dichotomized as (i) low; and (ii) normal birthweight. Low birthweight was defined as a weight below 2500 g (World Health Organization).

##### 2.3.2.5 Indicators of proximal processes (Process)

Four questions from the 3-years-old questionnaire were operationalized as indicators of proximal processes. Shared book-reading; and how often the child was meeting relatives, friends, or acquaintances of the family were categorized as follows: (i) every day; (ii) a few times a week; (iii) once a week or less often. Sedentary screentime during the week (i.e., on weekdays), and sedentary screentime during the weekend were dichotomized as (i) ≤ 1 h per day; and (ii) > 1 h per day (World Health Organization, 2019).

One item in the questionnaire concerned preschool attendance: “Is your child in childcare?“. However, it was regarded as having too little discriminatory power, as almost all children of that age attend childcare in Sweden, and therefore it was not used as an indicator of proximal processes.

### 2.4 Statistical analysis

#### 2.4.1 Descriptive statistics

The proportion of children above the cut-off points of 50 and 59 for each of the variables described above is displayed in Table 1, with p-values obtained from Pearson's chi2.

**Table 1:** Children with a high ASQ:SE score, for each variable relating to family context, personal characteristics, and proximal processes (N = 6033).

	Total	Children with an ASQ:SE score ≥50, n (%) n = 923 (15.3)	p-value*	Children with an ASQ:SE score ≥59, n (%) n = 532 (8.8)	p-value*
CONTEXT					
**Parents' place of birth**	6000		<**0.001**		<**0.001**
Both born in Sweden	5264	753 (14.3)		427 (8.1)	
One born in Sweden	604	123 (20.4)		73 (12.1)	
Both born outside Sweden	132	42 (31.8)		28 (21.2)	
**Parental education**	6030		<**0.001**		<**0.001**
Both more than high-school	2072	275 (13.3)		161 (7.8)	
One more than high-school	2012	289 (14.4)		151 (7.5)	
Neither more than high-school	1946	359 (18.4)		220 (11.3)	
**Family Income**	6032		<**0.001**		<**0.001**
1 (highest quintile)	1206	150 (12.4)		85 (7.0)	
2	1206	183 (15.2)		108 (9.0)	
3	1207	177 (14.7)		95 (7.9)	
4	1206	168 (13.9)		95 (7.9)	
5 (lowest quintile)	1207	245 (20.3)		149 (12.3)	
**Living arrangement, both parents living together**	5942		**0.004**		**0.003**
Yes	5543	824 (14.9)		468 (8.4)	
No	399	81 (20.3)		51 (12.8)	
**Parents' alcohol habits**	6015		<**0.001**		**0.001**
Neither parent with at-risk use or addiction	5358	780 (14.6)		448 (8.4)	
One or both parents with at-risk use or addiction	657	139 (21.2)		81 (12.3)	
**Parents' smoking habits**	5326		<**0.001**		<**0.001**
Neither parent smokes	4789	683 (14.3)		383 (8.0)	
One or both of the parents smoke	537	135 (25.1)		96 (17.9)	
**Parents' drug use**	6010		<**0.001**		**0.001**
Neither ever used drugs	5025	725 (14.4)		417 (8.3)	
One used drugs once or more	773	145 (18.8)		81 (10.5)	
Both used drugs once or more	212	49 (23.1)		31 (14.6)	
**Parental stress**	5999		<**0.001**		<**0.001**
Neither parent feels stressed a lot	4765	685 (14.4)		379 (8.0)	
One or both parents feel stressed a lot	1234	234 (19.0)		151 (12.2)	
**Mental illness and/or disorder (mother)**	6033		<**0.001**		<**0.001**
No	5551	793 (14.3)		453 (8.2)	
Yes	482	130 (27.0)		79 (16.4)	
**Mental illness and/or disorder (partner)**	6033		<**0.001**		<**0.001**
No	5787	865 (14.9)		494 (8.5)	
Yes	246	58 (23.6)		38 (15.4)	
PERSON					
**Gestational age**	5391		**0.028**		**0.030**
Normal	5110	754 (14.8)		428 (8.4)	
Preterm	281	55 (19.6)		34 (12.1)	
**Birthweight**	5386		0.380		0.158
Normal	5182	773 (14.9)		438 (8.5)	
Low birthweight	204	35 (17.2)		23 (11.3)	
**Sex**	6033		<**0.001**		<**0.001**
Boys	3102	600 (19.3)		361 (11.6)	
Girls	2931	323 (11.0)		171 (5.8)	
PROCESS					
**Child meets relatives, friends, family acquaintances**	5839		<**0.001**		<**0.001**
Every day	595	92 (15.5)		45 (7.6)	
A few times a week	3634	511 (14.1)		292 (8.0)	
Once a week or less often	1610	298 (18.5)		181 (11.2)	
**Shared book-reading**	6005		<**0.001**		<**0.001**
Every day	3938	538 (13.7)		301 (7.6)	
A few times a week	1624	272 (16.7)		160 (9.9)	
Once a week or less often	443	110 (24.8)		70 (15.8)	
**Child sedentary screen time during the week**	5777		<**0.001**		<**0.001**
≤1 h	3792	488 (12.9)		267 (7.0)	
>1 h	1985	390 (19.6)		239 (12.0)	
**Child sedentary screen time during the weekend**	5837		<**0.001**		<**0.001**
≤1 h	1760	204 (11.6)		101 (5.7)	
>1 h	4077	680 (16.7)		407 (10.0)	

*p-values were obtained from Pearson's chi2.

#### 2.4.2 Logistic regression models

**Fig. 2:**
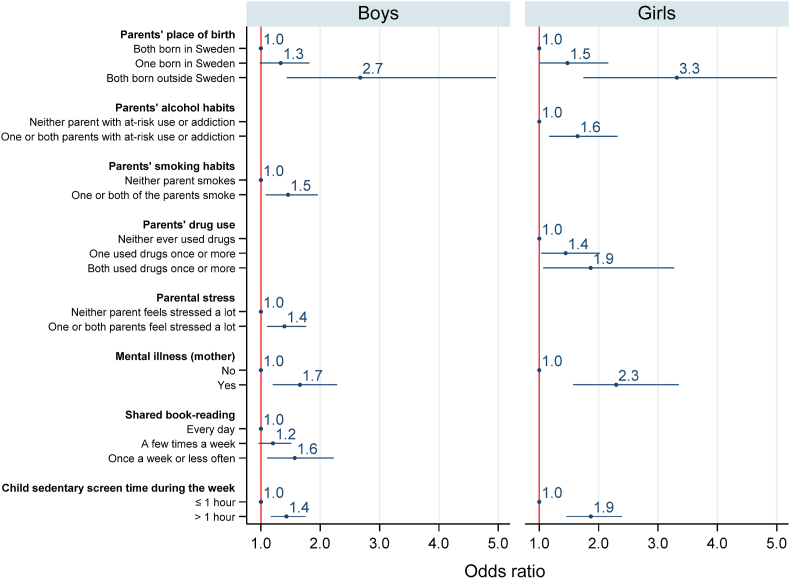
Odds ratios of being above the ASQ:SE cut-off of 50 for boys (n = 2574) and girls (n = 2770). Results from backward elimination, with 95% CI. Variables were manually removed one by one, starting with the one with the highest p-value. Backward selection stopped when only variables with p-values ≤0.05 remained in the model, except for parents' place of birth, parental education, and family income, which were always included. Only variables with odds ratios with a significant p-value are displayed.

Logistic regressions to assess whether the independent variables were associated with a high ASQ:SE score (≥50) were performed for boys and girls separately, as existing evidence from the same population indicated sex differences in social-emotional health (Eurenius et al., 2019; Vaezghasemi et al., 2020). Variables were entered in the logistic model as blocks, corresponding to the groupings of variables described above. Blocks of variables relating to the family social context were entered first. Parents' lifestyle variables were added in the second model, and indicators of parents' mental illness were incorporated in the third. Children's personal characteristics were then added in the fourth model and indicators of proximal processes were entered in the fifth (Table 2, Table 3). This approach was used to demonstrate how different blocks of theory-driven variables (context, person, and process) are independently associated with the outcome of interest. Finally, a backward selection of variables adjusted for parents' place of birth, parental education, and family income; and based on the statistical significance of the other variables (p ≤ 0.05) resulted in a final model (Fig. 2).

**Table 2:** Results from multiple logistic regressions displaying the odds ratios of having a high ASQ:SE score (above 50) for 3-year-olds boys.

	Context						Person		Process	
	Family social context	p-value	Parents' lifestyle	p-value	Parents' mental health	p-value	Child characteristics	p-value	Proximal processes	p-value
	(n = 3027)		(n = 2649)		(n = 2649)		(n = 2355)		(n = 2182)	
CONTEXT										
**Parents' place of birth**										
Both born in Sweden	1		1		1		1		1	
One born in Sweden	1.44	**0.012**	1.29	0.104	1.30	0.089	1.16	0.393	1.11	0.578
Both born outside Sweden	2.53	<**0.001**	2.70	**0.001**	2.84	**0.001**	3.20	<**0.001**	3.38	**0.001**
**Parental education**										
Both more than high-school	1		1		1		1		1	
One more than high-school	1.17	0.183	1.22	0.113	1.19	0.164	1.17	0.267	1.15	0.336
Neither more than high-school	1.43	**0.002**	1.44	**0.005**	1.39	**0.012**	1.44	**0.009**	1.52	**0.006**
**Family Income**										
1 (highest quintile)	1		1		1		1		1	
2	1.15	0.383	1.17	0.362	1.18	0.327	1.16	0.415	1.16	0.447
3	1.20	0.232	1.21	0.249	1.22	0.220	1.25	0.218	1.23	0.265
4	1.11	0.508	1.05	0.779	1.04	0.803	1.08	0.689	1.02	0.910
5 (lowest quintile)	1.47	**0.014**	1.46	**0.024**	1.42	**0.036**	1.37	0.080	1.24	0.273
**Living arrangement, both parents living together**										
Yes	1		1		1		1		1	
No	1.03	0.854	0.90	0.599	0.82	0.317	0.77	0.219	0.78	0.295
**Parents' alcohol habits**										
Neither parent with at-risk use or addiction			1		1		1		1	
One or both parents with at-risk use or addiction			1.25	0.155	1.24	0.175	1.23	0.201	1.15	0.424
**Parents' smoking habits**										
Neither parent smokes			1		1		1		1	
One or both of the parents smoke			1.52	**0.006**	1.48	**0.010**	1.59	**0.004**	1.51	**0.015**
**Parents' drug use**										
Neither ever used drugs			1		1		1		1	
One used drugs once or more			1.07	0.634	1.02	0.911	1.04	0.814	1.03	0.858
Both used drugs once or more			1.45	0.117	1.39	0.172	1.56	0.082	1.73	**0.042**
**Parental stress**										
Neither parent feels stressed a lot			1		1		1		1	
One or both parents feel stressed a lot			1.39	**0.004**	1.34	**0.013**	1.25	0.081	1.33	**0.032**
**Mental illness (mother)**										
No					1		1		1	
Yes					1.76	**0.001**	1.89	<**0.001**	1.81	**0.001**
**Mental illness (partner)**										
No					1		1		1	
Yes					1.13	0.602	1.00	0.997	1.03	0.924
PERSON										
**Gestational age**										
Normal							1		1	
Preterm							1.47	0.181	1.35	0.318
**Birthweight**										
Normal							1		1	
Low birthweight							0.96	0.921	1.19	0.653
PROCESS										
**Child meets relatives, friends, family acquaintances**										
Every day									1	
A few times a week									0.87	0.472
Once a week or less often									1.22	0.317
**Shared book-reading**										
Every day									1	
A few times a week									1.18	0.198
Once a week or less often									1.50	**0.036**
**Child sedentary screen time during the week**										
≤1 h									1	
>1 h									1.39	**0.008**
**Child sedentary screen time during the weekend**										
≤1 h									1	
>1 h									1.22	0.151

AU-ROC (with 95% CI)	0.5807 [0.55475–0.60655]		0.6086 [0.58154–0.63572]		0.6160 [0.58877–0.64317]		0.6232 [0.59391–0.65251]		0.6559 [0.62641–0.68529]

**Table 3:** Results from multiple logistic regressions displaying the odds ratios of having a high ASQ:SE score (above 50) for 3-year-olds girls.

	Context						Person		Process	
	Family social context	p-value	Parents' lifestyle	p-value	Parents' mental health	p-value	Child characteristics	p-value	Proximal processes	p-value
	(n = 2881)		(n = 2542)		(n = 2542)		(n = 2259)		(n = 2074)	
CONTEXT										
**Parents' place of birth**										
Both born in Sweden	1		1		1		1		1	
One born in Sweden	1.36	0.093	1.30	0.196	1.35	0.137	1.32	0.205	1.21	0.427
Both born outside Sweden	2.79	**0.001**	2.89	**0.002**	3.12	**0.001**	2.96	**0.003**	2.18	0.055
**Parental education**										
Both more than high-school	1		1		1		1		1	
One more than high-school	0.94	0.672	0.80	0.194	0.80	0.190	0.77	0.162	0.83	0.329
Neither more than high-school	1.43	**0.016**	1.35	0.058	1.29	0.114	1.26	0.181	1.21	0.331
**Family Income (quintiles)**										
1 (highest quintile)	1		1		1		1		1	
2	1.36	0.103	1.51	**0.038**	1.46	0.058	1.41	0.122	1.42	0.132
3	0.92	0.703	1.01	0.962	0.99	0.964	1.08	0.754	0.92	0.739
4	0.89	0.559	0.78	0.284	0.73	0.169	0.76	0.269	0.72	0.198
5 (lowest quintile)	1.37	0.111	1.46	0.073	1.31	0.214	1.30	0.261	1.28	0.326
**Living arrangement, both parents living together**										
Yes	1		1		1		1		1	
No	1.41	0.120	0.97	0.907	0.82	0.466	0.86	0.606	0.76	0.400
**Parents' alcohol habits**										
Neither parent with at-risk use or addiction			1		1		1		1	
One or both parents with at-risk use or addiction			1.56	**0.014**	1.55	**0.017**	1.74	**0.004**	1.60	**0.022**
**Parents' smoking habits**										
Neither parent smokes			1		1		1		1	
One or both of the parents smoke			1.35	0.122	1.31	0.172	1.13	0.584	1.19	0.453
**Parents' drug use**										
Neither ever used drugs			1		1		1		1	
One used drugs once or more			1.53	**0.014**	1.46	**0.031**	1.36	0.114	1.45	0.071
Both used drugs once or more			2.03	**0.011**	1.70	0.062	1.91	**0.027**	2.31	**0.007**
**Parental stress**										
Neither parent feels stressed a lot			1		1		1		1	
One or both parents feel stressed a lot			1.27	0.116	1.21	0.218	1.29	0.125	1.16	0.395
**Mental illness (mother)**										
No					1		1		1	
Yes					2.20	<**0.001**	2.31	<**0.001**	2.52	<**0.001**
**Mental illness (partner)**										
No					1		1		1	
Yes					1.47	0.184	1.46	0.219	1.08	0.824
PERSON										
**Gestational age**										
Normal							1		1	
Preterm							1.65	0.199	1.61	0.270
**Birthweight**										
Normal							1		1	
Low birthweight							0.63	0.319	0.66	0.405
PROCESS										
**Child meets relatives, friends, family acquaintances**										
Every day									1	
A few times a week									1.23	0.473
Once a week or less often									1.70	0.079
**Shared book-reading**										
Every day									1	
A few times a week									1.01	0.948
Once a week or less often									1.82	**0.020**
**Child sedentary screen time during the week**										
≤1 h									1	
>1 h									1.88	<**0.001**
**Child sedentary screen time during the weekend**										
≤1 h									1	
>1 h									1.09	0.642

AU-ROC (with 95% CI)	0.5984 [0.56562–0.63122]		0.6391 [0.60425–0.67398]		0.6526 [0.61767–0.68759]		0.6588 [0.62142–0.69622]		0.6967 [0.65858–0.73487]

The Area Under the Receiver Operating Characteristic curve (AU-ROC) (Lemeshow et al., 2013) and its corresponding Confidence Interval (CI) were computed to evaluate how well the variables included in the model discriminated between children identified as having social-emotional problems and children not identified as having social-emotional problems. AU-ROC results are bounded between 0.5 (no discrimination) and 1 (perfect discrimination). Current work in social epidemiology differentiates AU-ROC discrimination in the following way: AU-ROC = 0.5–0.6: ‘absent or very small discrimination of the model’; AU-ROC >0.6–≤0.7: ‘moderate’; AU-ROC >0.7–≤0.8: ‘large’; AU-ROC >0.8: ‘very large’ (Axelsson Fisk et al., 2021).

#### 2.4.3 Additional analysis

The five logistic regression models described above were also repeated for an ASQ:SE score categorized as (i) ≥59; and (ii) < 59 (Supplement: Tables 4 and 5). The robustness of the main results to the way of coding the common variables encompassing both parents was also evaluated through complete case analysis, by running logistic regressions with only those children whose parents had all information available on education level and lifestyle (Supplement: Tables 6 and 7).

All analysis were performed using Stata/BE version 17.0 (StataCorp, College Station, Texas 77,845 US).

### 2.5 Ethics

All participants provided written informed consent in conjunction with each questionnaire. It explicitly included the collection of additional individual-level data from national registers and the linking of family members. In the case of the 3-year-olds, consent was provided by one of the parents or by another legal guardian. The study was approved by the Regional Ethical Review Board in Umeå (2013-268-31Ö and 2017/401-32).

## 3 Results

### 3.1 Descriptive results

In this sample, 923 children (15.3%) had an ASQ:SE score ≥50 and 532 (8.8%) had an ASQ:SE score ≥59, indicating social-emotional problems (Table 1). The prevalence of social-emotional problems in 3-years-old children as measured by the ASQ:SE was higher in families where parent(s) were not born in Sweden, where both parents had a lower educational level, or in families who belonged to the lowest income quintile. In addition, children with parents reporting higher levels of alcohol consumption, smoking, and stress during pregnancy; or previous drug use, tended to score more often above the ASQ:SE cut-off value, whether it was 50 or 59. The same pattern was seen for children whose parent(s) had accessed specialist healthcare for a mental illness. The prevalence of social-emotional problems was also higher for boys, in children born preterm, and in those born with a low weight (but was not significant for low birthweight). In addition, children who met less frequently with acquaintances, had less frequent shared book-reading, and had more than 1 h of daily screentime were more often scoring above either cut-off.

### 3.2 Logistic regression models

Overall, less favourable family social context, detrimental lifestyle of the parents during pregnancy, and mental illness in parents were associated with higher odds of social-emotional problems in 3-year-olds (Table 2, Table 3). Conversely, children's characteristics at birth were not significantly associated with social-emotional problems at age 3. Unfavourable proximal processes were associated with higher odds of social-emotional problems, adjusted for family social context, parents' lifestyle and mental illness diagnostic, and characteristics at birth. Some similarities were observed between boys and girls in logistic regressions. However, some variables were significantly associated with higher odds of social-emotional problems only for boys, and some only for girls.

#### 3.2.1 Family social context

Having both parents born outside Sweden was significantly associated with higher odds of social-emotional problems in boys (OR 2.53, p < 0.001) and in girls (OR 2.79, p = 0.001) in the first regression model; compared to having both parents born in Sweden. This relationship remained significant for boys across models, but not for girls in the final one, which also included proximal processes. Similarly, children whose parents were high-school educated at most were at higher odds of social-emotional problems in the first regression (Boys: OR 1.43, p = 0.002 | Girls: OR 1.43, p = 0.016). Although this remained significant for boys throughout models, this was again not the case for girls. Boys whose family income was in the lowest quintile were at greater odds of social-emotional problems than boys whose family income was in the highest quintile in the first model (OR 1.47; p = 0.014). However, the association did not remain significant across models. No significant association of this kind was observed for girls.

#### 3.2.2 Parents’ lifestyle and mental illness

Different patterns of association between parents’ lifestyle during pregnancy and social-emotional problems at age 3 were observed for boys and girls. Having at least one parent with at-risk alcohol use (Second model: OR 1.56, p = 0.014) or both parents who used drugs in the past (Second model: OR 2.03, p = 0.11) was significantly associated with higher odds of social-emotional problems in girls across models compared to the reference categories. Conversely, boys with at least one parent who smoked (Second model: OR 1.52, p = 0.006) or felt stressed a lot (Second model: OR 1.39; p = 0.004) were more likely to experience social-emotional problems.

Boys and girls shared a similar pattern when it came to parents' mental illness. Children whose mother had accessed specialist healthcare for a mental illness were at significantly higher risk of social-emotional problems compared to children with a mother without such diagnostic (Third model; Boys: OR 1.76, p = 0.001 | Girls: OR 2.20, p < 0.001). On the other hand, partner's mental illness was not significantly associated with the outcome.

#### 3.2.3 Personal characteristics

In the fourth model, gestational age and birthweight did not display any significant association with social-emotional problems, and this did not change in the fifth model.

#### 3.2.4 Indicators of proximal processes

Shared book-reading taking place once a week or less often was associated with greater odds of social-emotional problems in boys (OR 1.50, p = 0.036) and in girls (OR 1.82, p = 0.020) compared to shared book-reading every day, adjusted for all variables relating to family social context, parents’ lifestyle and mental health, and characteristics at birth. A similar pattern was observed for those children exposed to more than 1 h of screentime a day during the week (Boys: OR 1.39, p = 0.008 | Girls: OR 1.88, p < 0.001) compared to 1 h or less. However, the other variables relating to proximal processes were not significantly associated with social-emotional problems in children.

#### 3.2.5 Area under the Receiver Operating Characteristic curve

The AU-ROC for the fifth model was 0.66 for boys (CI: [0.63–0.69]) and 0.70 for girls (CI: [0.66–0.73]), indicating that the models had a moderate capacity to discriminate between children above and below the cut-off value of ≥50.

#### 3.2.6 Backward selection of variables

After backward selection adjusted for parents’ place of birth, parental education, and family income, higher odds of social-emotional problems were observed in boys: with both parents born outside Sweden (OR 2.67, p = 0.002), with one or both parents smoking during pregnancy (OR 1.46, p = 0.013), with one or both parents feeling stressed a lot during pregnancy (OR 1.40, p = 0.005), whose mothers had accessed specialist healthcare for a mental illness (OR 1.66, p = 0.002), for whom shared book-reading took place once a week or less often (OR 1.57, p = 0.011), or with a sedentary screentime greater than 1 h a day on weekdays (OR 1.43, p = 0.001) (Fig. 2).

For girls, greater risk of social-emotional problems was found for those: with one (OR 1.48, p = 0.045) or both parents born outside Sweden (OR 3.32, p < 0.001), with one or both parents with at-risk alcohol use (OR 1.65, p = 0.004), with one (OR 1.44, p = 0.032) or both (OR 1.87, p = 0.030) parents who used drugs once or more, whose mother had a mental illness diagnostic (OR 2.29, p < 0.001), or with a screentime higher than 1 h a day on weekdays (OR 1.87, p < 0.001).

### 3.3 Additional analysis

#### 3.3.1 Logistic regression models for the cut-off of ≥59

Additional analysis with a cut-off value of ≥59 for the outcome yielded similar results overall for boys and for girls (Supplement: Tables 4 and 5). The following differences were noted for boys. The lowest income quintile was not significantly associated with higher odds of social-emotional problems in boys in the additional analysis (First model; OR 1.34, p = 0.121). Likewise, although the direction of effect was similar, the association between shared book-reading and social-emotional problems did not reach statistical significance (OR 1.55, p = 0.061 for “once a week or less often”). Conversely, having more than 1 h of screentime a day on weekends (OR 1.48, p = 0.034) was significantly associated with greater risk of social-emotional problems.

The differences that were observed for girls related mostly to parents’ lifestyle during pregnancy. In the second and third models with a cut-off value of ≥59, girls having at least one parent who smoked were found to be significantly more likely to experience social-emotional problems compared to girls with non-smoking parents (Second model; OR 1.74, p = 0.018). And although the direction of the effect was similar, girls having at least one parent with at-risk alcohol use were not at significantly greater risk of social-emotional problems compared to the reference category (Second model; OR 1.57, p = 0.053).

The AU-ROC for the fifth model was 0.69 for boys (CI: [0.65–0.72]) and 0.74 for girls (CI: [0.69–0.79]), indicating that the models had a moderate capacity to discriminate between boys, and a large capacity to discriminate between girls above and below the cut-off value of ≥59.

#### 3.3.2 Complete case analysis for the cut-off of ≥50

The analysis with a sample restricted to only those children whose parents had all information available on education level and lifestyle also returned similar results overall, both for boys and girls (Supplement: Tables 6 and 7). However, some differences were observed with the main analysis. For example, boys with parents having at most completed high-school were not at significantly greater odds of social-emotional problems compared to boys whose both parents were more than high-school educated (First model; OR 1.16, P = 0.405). The same was seen for boys regarding shared book-reading (OR 1.27, p = 0.381).

For girls, the lowest parental education category (First model; OR 1.31, p = 0.196) and having parent(s) with at-risk alcohol use (Second model; OR 1.53, p = 0.092) was not significantly associated with higher odds of social-emotional problems, although the direction of effect was the same as in the main analysis.

## 4 Discussion

From pregnancy, to birth, and to 3 years of age, we found that determinants of children's social-emotional problems were multifaceted and varied in how distal or proximal they were to the children. Children growing up in unfavourable family social contexts were more likely to experience social-emotional problems at age 3. Similarly, detrimental parents' lifestyle during pregnancy were associated with greater risk of social-emotional vulnerability. Importantly, we also assessed associations between proximal processes and social-emotional problems, adjusted for all other variables. Among our four indicators, low frequency of shared book-reading and longer screentime on weekdays were most consistently related to social-emotional problems. We also observed that relevant factors sometimes differed for boys and girls.

The role of socio-economic circumstances in shaping child mental health has been well established (Pillas et al., 2014; Reiss, 2013). Despite Sweden being a high-income country with a welfare state, our results do not contradict previous research in this area, although the only social factor strongly associated with social-emotional problems across models was having both parents born outside Sweden. Similarly, the role that parents' lifestyle during pregnancy may play on children's social-emotional-health has been investigated elsewhere (Dolan et al., 2016; Easey et al., 2019; Lai et al., 2020; Lee et al., 2020) and our results indeed suggest that they are of importance. Our results on maternal mental illness are in accordance with previous research that indicated a relationship between mother's and child's mental health (Goodman et al., 2011; Lai et al., 2020; Weissman et al., 2006), but we did not find such relationship with mental illness in the partner. Unlike in other studies (Cheong et al., 2017; McIntosh et al., 2021; Olsen et al., 2022), no significant relationship between children's characteristics at birth and social-emotional problems was observed.

Our results highlight the relevance of proximal processes in studying developmental outcomes, as highlighted by Bronfenbrenner's theory (Bronfenbrenner, 2005a). Other studies have found that parent-child joint attention and book-reading contributed to vocabulary development in early childhood (Farrant & Zubrick, 2011), and that intervening to improve parenting through parent-child reading and playing had a positive effect on children's social-emotional development (Mendelsohn et al., 2018). In our study, shared book-reading once a week or less often was significantly associated with greater odds of social-emotional problems for boys in the main analysis, and for girls in all analyses. This is consistent with results from a longitudinal study conducted in the United States, which found that a lower frequency of shared book-reading was associated with subsequent social-emotional problems (Martin et al., 2021). We also found that higher sedentary screentime on weekdays, a form of interaction between children and objects and symbols, was associated with higher odds of social-emotional vulnerability across all analysis for boys and girls. Those results are similar to findings from a Canadian longitudinal study (Madigan et al., 2019).

The finding that the associations between the outcome and independent variables differed by sex lends support to the hypothesis that personal characteristics can moderate relations between context, proximal processes, and outcomes (Bronfenbrenner, 2005a). For example, we found that parents' alcohol habits were significantly associated with social-emotional problems in girls, but not in boys. Such a moderating role of personal characteristics has previously been observed in studies drawing from Bronfenbrenner's theory and investigating mental health related outcomes (Behnke et al., 2010; Votruba-Drzal et al., 2010), although not consistently (Benson & Buehler, 2012).

### 4.1 Relevance for public health policy

This study justifies ongoing efforts to support healthy lifestyles and the mental health of parents-to-be in Antenatal Care. Parents' lifestyle and mental health could also be potential targets for further health promotion to enhance the social-emotional health of children. For example, it has been suggested that cognitive-behavioural therapy and psychoeducation could be used in interventions aimed at preventing mental disorders in children of parents with mental illness (Lannes et al., 2021). In addition, results regarding indicators of proximal processes could also encourage directing future efforts towards positive parenting promotion. Implementing this kind of program in paediatric primary care has been suggested to be effective in improving children's social-emotional development (Mendelsohn et al., 2018). The Triple P program, implemented in Uppsala municipality in Sweden, is an example of such an intervention aiming to improve positive parenting in a population (Dahlberg et al., 2022).

Thus, overall, our study speaks to the relevance of Bronfenbrenner's process-person-context-time theory in public health, and partly addresses queries surrounding its usefulness and the adequacy of considering proximal processes in public mental health research (Eriksson et al., 2018). This theory might be useful to guide public health interventions aimed at improving children's social-emotional health. It allows researchers and policy-makers to consider different targets for intervention, both proximal – such as shared book-reading – and distal – such as educational inequalities, as well as their interrelations.

Finally, the moderate discriminatory accuracy of the full models indicates that although certain groups of children are at higher risk of social-emotional problems, universal interventions might be a more appropriate response than targeted interventions since the heterogeneity within each at-risk group might be large. This should not, however, deter policy-makers from implementing interventions favouring the least advantaged even if such interventions are universal, in accordance with principles of proportionate universalism (Marmot & Bell, 2012).

### 4.2 Methodological considerations

This study assessed the association between numerous indicators of family context, person characteristics and proximal processes with social-emotional problems at 3 years of age. Although the number of indicators investigated is a strength of the study, several limitations should be acknowledged.

Due to linkage from several sources, part of the initial sample has been lost, similarly to previous studies on total population in this context. However, because the prevalence of social-emotional problems stayed similar when reducing the sample size, the direction of the results might be unaffected, and we do not expect this to result in selection bias. Also, since children were only considered for the analysis when the 3-year-old questionnaire was linked to at least one questionnaire filled out during pregnancy by one of the parents, no children born outside of Sweden were included.

In addition, the ASQ:SE being a parent-completed questionnaire, child social-emotional health might be reported with bias. Parents’ reports on their child can be affected by their own stress, mental illness (Godoy et al., 2014), and by their child gender (Najman et al., 2001). Differences between boys and girls could then partly arise from the different expectations that parents have for them (Bayly & Gartstein, 2013), and reporting a high-score on the ASQ:SE instrument could also indicate help-seeking from parents with stress and mental illness (Godoy et al., 2014). Furthermore, this study used other self-reported questionnaires, and reporting bias on independent variables might therefore be affecting the results. Self-reported indicators include living arrangement, those related to family lifestyle, and to proximal processes. However, variables operationalized from register data are not affected by this issue, which is a strength of this study.

Although variables related to family context and to personal characteristics were obtained prior to the filling out of the 3-years-old questionnaire, indicators of proximal processes and the outcome were measured at the same point in time. Therefore, reverse causation or feedback cannot be excluded. A child with social-emotional problems could be less prone to shared book reading to begin with. The parents might then read to the child less often, and the occurrence of this proximal process would then partially be the consequence of the child's social-emotional health. Similarly, high screen time could be explained by already existing social-emotional vulnerabilities in children. In addition, because parents' lifestyle was measured during pregnancy, it is unknown what the associations would have been if they had been measured at another time.

Variables relating to the family were chosen for social context and parents' lifestyle, instead of separately for mothers and partners. Choosing variables characterising the family might give a better idea of the overall environment in which children develop. However, future research could distinguish between mothers and partners characteristics, as it might reveal different associations with children's social-emotional health.

The use of self-reporting to assess proximal processes means that we could not include a dimension of microtime in the study, as we could not directly observe those proximal processes while they happen, as was done elsewhere (Tudge et al., 2003). However, the questions relating to processes allowed us to evaluate how they occur over the mesotime (Bronfenbrenner & Morris, 1998). For example, we could evaluate the frequency of occurrence of shared book-reading over a week. Regarding macrotime, the study period was too short to investigate any intergenerational change or evolutions in society, as children's questionnaires were collected from 2014 to 2018. Future research could consider the impact of the COVID 19 pandemic on children's social-emotional health in Västerbotten, thereby including a dimension of macrotime.

Given the considerations above, and differences of Västerbotten and Sweden with other settings, it is unknown whether the results presented here can be generalized elsewhere. For example, we noted that there are social-emotional health inequalities based on social contexts in which children develop (Pillas et al., 2014; Reiss, 2013). Because social contexts vary from country to country, the associations observed might differ as well from country to country. In addition, other variables such as preschool attendance might be of greater interest in other settings. As was noted above, Sweden has a very high degree of preschool attendance among 1 to 5-year-olds, related to the high level of female gainful employment and low preschool fees in international comparison. However, children from families where both parents were born outside of Sweden have considerably lower levels of preschool attendance than children from families with at least one Swedish-born parent – a difference of 8–10 percent units (Swedish National Agency for Education, 2012). In the present study, the number of 3-year-old children with foreign background who were not attending preschool was too low to allow for an analysis. In this section, we mainly focused on discussing the determinants of social-emotional vulnerability and compared our finding with other studies in the field. However, more information on the comparison of the distribution and prevalence of social-emotional vulnerability can be found elsewhere (Eurenius et al., 2019; Vaezghasemi et al., 2020; Vezghasemi et al., 2022).

## 5 Conclusions

By considering a holistic approach, our findings suggest that determinants of social-emotional problems may include diverse factors located at a varying proximity to the 3-year-old children. The multifaceted aspect of those determinants also points to a range of possible interventions to improve children's social-emotional health. The targets of such interventions could go from healthy lifestyle promotion to supporting shared-book reading. Some of those possible interventions are already in place in Västerbotten – in Antenatal and Child Health Care as part of the Salut Programme – or elsewhere. The results of this study also highlight the potential relevance of the process-person-context-time theory for public health researchers and policy-makers, that could point to ways of improving existing health promotion efforts, of identifying additional targets for prevention, and in designing future interventions.

## Role of the funding source

This study was funded by Forte (grant 2021-00155). The funder had no involvement in the conduct of the research and in the preparation of the article.

## Ethical statement

All participants provided written informed consent in conjunction with each questionnaire. It explicitly included the collection of additional individual-level data from national registers and the linking of family members. In the case of the 3-year-olds, consent was provided by one of the parents or by another legal guardian. The study was approved by the Regional Ethical Review Board in Umeå (2013-268-31Ö and 2017/401-32).

## Credit author statement

**Masoud Vaezghasemi**: Conceptualization, Supervision, Data curation, Methodology, Formal analysis, Writing - review & editing; **Thomas Vogt**: Data curation, Methodology, Formal analysis, Writing - original draft, Writing - review & editing; **Marie Lindkvist**: Methodology, Formal analysis, Writing - review & editing; **Anni-Maria Pulkki-Brännström**: Writing - review & editing; **Linda Richter Sundberg**: Writing - review & editing; **Lisbeth Lundahl**: Writing - review & editing; **Sven-Arne Silfverdal**: Writing - review & editing; **Inna Feldman**: Writing - review & editing; **Anneli Ivarsson**: Conceptualization, Resources, Methodology, Writing - review & editing, Funding acquisition.

## Declaration of competing interest

None.

## Data Availability

The authors do not have permission to share data.

## Abbreviations

ASQ:SE: (Ages and Stages Questionnaires: Social-Emotional)
AU-ROC: (Area Under the Receiver Operating Characteristic curve)
CI: (Confidence Interval)
