# Supplementary material for: Multifaceted determinants of social-emotional problems in preschool children in Sweden: An ecological systems theory approach
Source: SSM Popul Health. 2023 Jan 21;21:101345. doi: 10.1016/j.ssmph.2023.101345 (PMC9918800; doi:10.1016/j.ssmph.2023.101345)
Supplement: Multimedia component 1 [file mmc1.docx]

**Online supplement to: multifaceted determinants of social-emotional problems in preschool children in Sweden: an ecological systems theory approach.**

Table 4: Results from multiple logistic regressions displaying the odds ratios of having a high ASQ:SE score (above 59) for 3-year-olds boys.

|  | Context | | | | | | +Person | | +Process | |
| --- | --- | --- | --- | --- | --- | --- | --- | --- | --- | --- |
|  | Family social context  (n=3027) | p-value | + Parents' lifestyle  (n= 2649) | p-value | +Parents' mental health  (n=2649) | p-value | Child characteristics  (n=2355) | p-value | Proximal processes  (n=2182) | p-value |
| CONTEXT |  |  |  |  |  |  |  |  |  |  |
| **Parents' place of birth** |  |  |  |  |  |  |  |  |  |  |
| Both born in Sweden | 1 |  | 1 |  | 1 |  | 1 |  | 1 |  |
| One born in Sweden | 1.59 | **0.006** | 1.30 | 0.154 | 1.31 | 0.147 | 1.09 | 0.681 | 0.98 | 0.931 |
| Both born outside Sweden | 2.45 | **0.003** | 2.68 | **0.003** | 2.87 | **0.002** | 3.26 | **0.001** | 3.10 | **0.004** |
| **Parental education** |  |  |  |  |  |  |  |  |  |  |
| Both more than high-school | 1 |  | 1 |  | 1 |  | 1 |  | 1 |  |
| One more than high-school | 1.03 | 0.846 | 1.01 | 0.953 | 0.98 | 0.922 | 0.91 | 0.565 | 0.89 | 0.541 |
| Neither more than high-school | 1.35 | **0.035** | 1.27 | 0.134 | 1.23 | 0.199 | 1.19 | 0.307 | 1.23 | 0.266 |
| **Family Income (quintiles)** |  |  |  |  |  |  |  |  |  |  |
| 1 (highest quintile) | 1 |  | 1 |  | 1 |  | 1 |  | 1 |  |
| 2 | 1.11 | 0.588 | 1.04 | 0.833 | 1.05 | 0.809 | 1.02 | 0.914 | 0.98 | 0.937 |
| 3 | 0.96 | 0.826 | 0.91 | 0.652 | 0.92 | 0.673 | 0.87 | 0.539 | 0.85 | 0.484 |
| 4 | 1.04 | 0.834 | 0.96 | 0.840 | 0.94 | 0.764 | 0.92 | 0.706 | 0.84 | 0.472 |
| 5 (lowest quintile) | 1.34 | 0.121 | 1.30 | 0.190 | 1.25 | 0.266 | 1.17 | 0.467 | 1.00 | 0.992 |
| **Living arrangement, both parents living together:** |  |  |  |  |  |  |  |  |  |  |
| Yes | 1 |  | 1 |  | 1 |  | 1 |  | 1 |  |
| No | 1.17 | 0.458 | 0.99 | 0.967 | 0.87 | 0.561 | 0.83 | 0.466 | 0.91 | 0.742 |
| **Parents' alcohol habits** |  |  |  |  |  |  |  |  |  |  |
| Neither parent with at-risk use or addiction |  |  | 1 |  | 1 |  | 1 |  | 1 |  |
| One or both parents with at-risk use or addiction |  |  | 1.21 | 0.302 | 1.22 | 0.299 | 1.28 | 0.218 | 1.21 | 0.357 |
| **Parents' smoking habits** |  |  |  |  |  |  |  |  |  |  |
| Neither parent smokes |  |  | 1 |  | 1 |  | 1 |  | 1 |  |
| One or both of the parents smoke |  |  | 1.79 | **0.001** | 1.73 | **0.002** | 1.95 | **<0.001** | 1.90 | **0.001** |
| **Parents' drug use** |  |  |  |  |  |  |  |  |  |  |
| Neither ever used drugs |  |  | 1 |  | 1 |  | 1 |  | 1 |  |
| One used drugs once or more |  |  | 0.98 | 0.900 | 0.92 | 0.646 | 0.89 | 0.551 | 0.92 | 0.695 |
| Both used drugs once or more |  |  | 1.27 | 0.399 | 1.20 | 0.533 | 1.42 | 0.243 | 1.69 | 0.097 |
| **Parental stress** |  |  |  |  |  |  |  |  |  |  |
| Neither parent feels stressed a lot |  |  | 1 |  | 1 |  | 1 |  | 1 |  |
| One or both parents feel stressed a lot |  |  | 1.63 | **<0.001** | 1.56 | **0.001** | 1.48 | **0.009** | 1.68 | **0.001** |
| **Mental illness (mother)** |  |  |  |  |  |  |  |  |  |  |
| No |  |  |  |  | 1 |  | 1 |  | 1 |  |
| Yes |  |  |  |  | 1.51 | **0.036** | 1.76 | **0.006** | 1.66 | **0.019** |
| **Mental illness (partner)** |  |  |  |  |  |  |  |  |  |  |
| No |  |  |  |  | 1 |  | 1 |  | 1 |  |
| Yes |  |  |  |  | 1.64 | 0.051 | 1.58 | 0.091 | 1.55 | 0.136 |
|  |  |  |  |  |  |  |  |  |  |  |
| PERSON |  |  |  |  |  |  |  |  |  |  |
| **Gestational age** |  |  |  |  |  |  |  |  |  |  |
| Normal |  |  |  |  |  |  | 1 |  | 1 |  |
| Preterm |  |  |  |  |  |  | 1.27 | 0.504 | 1.33 | 0.437 |
| **Birthweight** |  |  |  |  |  |  |  |  |  |  |
| Normal |  |  |  |  |  |  | 1 |  | 1 |  |
| Low birthweight |  |  |  |  |  |  | 1.41 | 0.413 | 1.59 | 0.288 |
|  |  |  |  |  |  |  |  |  |  |  |
| PROCESS |  |  |  |  |  |  |  |  |  |  |
| **Child meets relatives, friends, family acquaintances** |  |  |  |  |  |  |  |  |  |  |
| Every day |  |  |  |  |  |  |  |  | 1 |  |
| A few times a week |  |  |  |  |  |  |  |  | 1.13 | 0.623 |
| Once a week or less often |  |  |  |  |  |  |  |  | 1.56 | 0.089 |
| **Shared book-reading** |  |  |  |  |  |  |  |  |  |  |
| Every day |  |  |  |  |  |  |  |  | 1 |  |
| A few times a week |  |  |  |  |  |  |  |  | 1.15 | 0.401 |
| Once a week or less often |  |  |  |  |  |  |  |  | 1.55 | 0.061 |
| **Child sedentary screen time during the week** |  |  |  |  |  |  |  |  |  |  |
| ≤ 1 hour |  |  |  |  |  |  |  |  | 1 |  |
| > 1 hour |  |  |  |  |  |  |  |  | 1.54 | **0.004** |
| **Child sedentary screen time during the weekend** |  |  |  |  |  |  |  |  |  |  |
| ≤ 1 hour |  |  |  |  |  |  |  |  | 1 |  |
| > 1 hour |  |  |  |  |  |  |  |  | 1.48 | **0.034** |
|  |  |  |  |  |  |  |  |  |  |  |
| AU-ROC (with 95% CI) | 0.5850 [0.55315- 0.61691] |  | 0.6273 [0.59390-0.66074] |  | 0.6361 [0.60271-0.66941] |  | 0.6446 [0.60804-0.68117] |  | 0.6869 [0.65055-0.72324] |  |

Table 5: Results from multiple logistic regressions displaying the odds ratios of having a high ASQ:SE score (above 59) for 3-year-olds girls.

|  | Context | | | | | | +Person | | +Process | |
| --- | --- | --- | --- | --- | --- | --- | --- | --- | --- | --- |
|  | Family social context  (n= 2881) | p-value | + Parents' lifestyle  (n= 2542) | p-value | +Parents' mental health  (n=2542) | p-value | Child characteristics  (n=2259) | p-value | Proximal  processes  (n=2074) | p-value |
| CONTEXT |  |  |  |  |  |  |  |  |  |  |
| **Parents' place of birth** |  |  |  |  |  |  |  |  |  |  |
| Both born in Sweden | 1 |  | 1 |  | 1 |  | 1 |  | 1 |  |
| One born in Sweden | 1.05 | 0.846 | 1.03 | 0.905 | 1.09 | 0.764 | 1.12 | 0.700 | 1.07 | 0.839 |
| Both born outside Sweden | 3.50 | **0.001** | 3.20 | **0.005** | 3.51 | **0.003** | 3.01 | **0.020** | 2.24 | 0.124 |
| **Parental education** |  |  |  |  |  |  |  |  |  |  |
| Both more than high-school | 1 |  | 1 |  | 1 |  | 1 |  | 1 |  |
| One more than high-school | 0.78 | 0.269 | 0.66 | 0.089 | 0.66 | 0.084 | 0.66 | 0.121 | 0.68 | 0.166 |
| Neither more than high-school | 1.74 | **0.005** | 1.61 | **0.024** | 1.52 | **0.049** | 1.52 | 0.068 | 1.54 | 0.089 |
| **Family Income (quintiles)** |  |  |  |  |  |  |  |  |  |  |
| 1 (highest quintile) | 1 |  | 1 |  | 1 |  | 1 |  | 1 |  |
| 2 | 1.64 | 0.058 | 2.12 | **0.009** | 2.01 | **0.015** | 1.73 | 0.084 | 1.60 | 0.152 |
| 3 | 1.10 | 0.746 | 1.38 | 0.297 | 1.35 | 0.336 | 1.46 | 0.249 | 1.16 | 0.669 |
| 4 | 0.91 | 0.749 | 1.00 | 0.995 | 0.92 | 0.800 | 0.90 | 0.766 | 0.78 | 0.500 |
| 5 (lowest quintile) | 1.53 | 0.119 | 1.87 | **0.036** | 1.63 | 0.107 | 1.74 | 0.091 | 1.58 | 0.183 |
| **Living arrangement, both parents living together:** |  |  |  |  |  |  |  |  |  |  |
| Yes | 1 |  | 1 |  | 1 |  | 1 |  | 1 |  |
| No | 1.41 | 0.231 | 1.14 | 0.679 | 0.96 | 0.890 | 0.97 | 0.935 | 0.87 | 0.733 |
| **Parents' alcohol habits** |  |  |  |  |  |  |  |  |  |  |
| Neither parent with at-risk use or addiction |  |  | 1 |  | 1 |  | 1 |  | 1 |  |
| One or both parents with at-risk use or addiction |  |  | 1.57 | 0.053 | 1.52 | 0.071 | 1.55 | 0.083 | 1.39 | 0.223 |
| **Parents' smoking habits** |  |  |  |  |  |  |  |  |  |  |
| Neither parent smokes |  |  | 1 |  | 1 |  | 1 |  | 1 |  |
| One or both of the parents smoke |  |  | 1.74 | **0.018** | 1.70 | **0.024** | 1.35 | 0.258 | 1.46 | 0.173 |
| **Parents' drug use** |  |  |  |  |  |  |  |  |  |  |
| Neither ever used drugs |  |  | 1 |  | 1 |  | 1 |  | 1 |  |
| One used drugs once or more |  |  | 1.37 | 0.171 | 1.29 | 0.275 | 1.22 | 0.456 | 1.24 | 0.440 |
| Both used drugs once or more |  |  | 2.58 | **0.004** | 2.14 | **0.027** | 2.38 | **0.014** | 2.81 | **0.007** |
| **Parental stress** |  |  |  |  |  |  |  |  |  |  |
| Neither parent feels stressed a lot |  |  | 1 |  | 1 |  | 1 |  | 1 |  |
| One or both parents feel stressed a lot |  |  | 1.22 | 0.321 | 1.15 | 0.500 | 1.24 | 0.319 | 1.12 | 0.629 |
| **Mental illness (mother)** |  |  |  |  |  |  |  |  |  |  |
| No |  |  |  |  | 1 |  | 1 |  | 1 |  |
| Yes |  |  |  |  | 2.49 | **<0.001** | 2.74 | **<0.001** | 2.98 | **<0.001** |
| **Mental illness (partner)** |  |  |  |  |  |  |  |  |  |  |
| No |  |  |  |  | 1 |  | 1 |  | 1 |  |
| Yes |  |  |  |  | 1.19 | 0.635 | 1.15 | 0.721 | 0.97 | 0.937 |
|  |  |  |  |  |  |  |  |  |  |  |
| PERSON |  |  |  |  |  |  |  |  |  |  |
| **Gestational age** |  |  |  |  |  |  |  |  |  |  |
| Normal |  |  |  |  |  |  | 1 |  | 1 |  |
| Preterm |  |  |  |  |  |  | 2.24 | 0.108 | 2.39 | 0.109 |
| **Birthweight** |  |  |  |  |  |  |  |  |  |  |
| Normal |  |  |  |  |  |  | 1 |  | 1 |  |
| Low birthweight |  |  |  |  |  |  | 0.19 | **0.045** | 0.10 | **0.034** |
|  |  |  |  |  |  |  |  |  |  |  |
| PROCESS |  |  |  |  |  |  |  |  |  |  |
| **Child meets relatives, friends, family acquaintances** |  |  |  |  |  |  |  |  |  |  |
| Every day |  |  |  |  |  |  |  |  | 1 |  |
| A few times a week |  |  |  |  |  |  |  |  | 1.31 | 0.498 |
| Once a week or less often |  |  |  |  |  |  |  |  | 1.86 | 0.135 |
| **Shared book-reading** |  |  |  |  |  |  |  |  |  |  |
| Every day |  |  |  |  |  |  |  |  | 1 |  |
| A few times a week |  |  |  |  |  |  |  |  | 1.16 | 0.540 |
| Once a week or less often |  |  |  |  |  |  |  |  | 2.80 | **0.001** |
| **Child sedentary screen time during the week** |  |  |  |  |  |  |  |  |  |  |
| ≤ 1 hour |  |  |  |  |  |  |  |  | 1 |  |
| > 1 hour |  |  |  |  |  |  |  |  | 1.57 | **0.036** |
| **Child sedentary screen time during the weekend** |  |  |  |  |  |  |  |  |  |  |
| ≤ 1 hour |  |  |  |  |  |  |  |  | 1 |  |
| > 1 hour |  |  |  |  |  |  |  |  | 1.25 | 0.386 |
|  |  |  |  |  |  |  |  |  |  |  |
| AU-ROC (with 95% CI) | 0.6301 [0.58612- 0.67417] |  | 0.6716 [0.62432- 0.71894] |  | 0.6857 [0.63853- 0.73285] |  | 0.6923 [0.64294-0.74169] |  | 0.7360 [0.68533-0.78671] |  |

Table 6: Results from multiple logistic regressions displaying the odds ratios of having a high ASQ:SE score (above 50) for 3-year-olds boys (complete case analysis).

|  | Context | | | | | | +Person | | +Process | |
| --- | --- | --- | --- | --- | --- | --- | --- | --- | --- | --- |
|  | Family social context  (n=1426) | p-value | + Parents' lifestyle  (n=1426) | p-value | +Parents' mental health  (n=1426) | p-value | Child characteristics  (n=1245) | p-value | Proximal processes  (n=1149) | p-value |
| CONTEXT |  |  |  |  |  |  |  |  |  |  |
| **Parents' place of birth** |  |  |  |  |  |  |  |  |  |  |
| Both born in Sweden | 1 |  | 1 |  | 1 |  | 1 |  | 1 |  |
| One born in Sweden | 1.46 | 0.073 | 1.36 | 0.150 | 1.38 | 0.139 | 1.25 | 0.355 | 1.17 | 0.553 |
| Both born outside Sweden | 3.56 | **0.005** | 3.10 | **0.014** | 3.18 | **0.012** | 3.93 | **0.004** | 5.21 | **0.002** |
| **Parental education** |  |  |  |  |  |  |  |  |  |  |
| Both more than high-school | 1 |  | 1 |  | 1 |  | 1 |  | 1 |  |
| One more than high-school | 1.09 | 0.600 | 1.15 | 0.416 | 1.12 | 0.530 | 1.02 | 0.926 | 0.98 | 0.909 |
| Neither more than high-school | 1.16 | 0.405 | 1.13 | 0.498 | 1.09 | 0.645 | 1.07 | 0.728 | 1.13 | 0.581 |
| **Family Income (quintiles)** |  |  |  |  |  |  |  |  |  |  |
| 1 (highest quintile) | 1 |  | 1 |  | 1 |  | 1 |  | 1 |  |
| 2 | 1.25 | 0.322 | 1.17 | 0.504 | 1.20 | 0.427 | 1.19 | 0.491 | 1.17 | 0.566 |
| 3 | 1.09 | 0.713 | 1.00 | 0.983 | 1.02 | 0.926 | 1.05 | 0.834 | 1.08 | 0.784 |
| 4 | 1.00 | 0.983 | 0.93 | 0.774 | 0.94 | 0.805 | 0.99 | 0.956 | 0.98 | 0.943 |
| 5 (lowest quintile) | 1.58 | **0.041** | 1.41 | 0.127 | 1.38 | 0.155 | 1.38 | 0.200 | 1.31 | 0.314 |
| **Living arrangement, both parents living together:** |  |  |  |  |  |  |  |  |  |  |
| Yes | 1 |  | 1 |  | 1 |  | 1 |  | 1 |  |
| No | 1.03 | 0.911 | 0.89 | 0.693 | 0.83 | 0.558 | 0.87 | 0.658 | 1.10 | 0.783 |
| **Parents' alcohol habits** |  |  |  |  |  |  |  |  |  |  |
| Neither parent with at-risk use or addiction |  |  | 1 |  | 1 |  | 1 |  | 1 |  |
| One or both parents with at-risk use or addiction |  |  | 0.86 | 0.528 | 0.85 | 0.494 | 0.80 | 0.374 | 0.76 | 0.308 |
| **Parents' smoking habits** |  |  |  |  |  |  |  |  |  |  |
| Neither parent smokes |  |  | 1 |  | 1 |  | 1 |  | 1 |  |
| One or both of the parents smoke |  |  | 1.65 | **0.012** | 1.59 | **0.020** | 1.68 | **0.015** | 1.51 | 0.065 |
| **Parents' drug use** |  |  |  |  |  |  |  |  |  |  |
| Neither ever used drugs |  |  | 1 |  | 1 |  | 1 |  | 1 |  |
| One used drugs once or more |  |  | 1.01 | 0.959 | 0.98 | 0.937 | 1.11 | 0.621 | 1.02 | 0.927 |
| Both used drugs once or more |  |  | 1.79 | **0.043** | 1.72 | 0.060 | 2.10 | **0.015** | 2.47 | **0.005** |
| **Parental stress** |  |  |  |  |  |  |  |  |  |  |
| Neither parent feels stressed a lot |  |  | 1 |  | 1 |  | 1 |  | 1 |  |
| One or both parents feel stressed a lot |  |  | 1.61 | **0.002** | 1.56 | **0.005** | 1.35 | 0.089 | 1.35 | 0.101 |
| **Mental illness (mother)** |  |  |  |  |  |  |  |  |  |  |
| No |  |  |  |  | 1 |  | 1 |  | 1 |  |
| Yes |  |  |  |  | 1.66 | **0.021** | 1.75 | **0.018** | 1.70 | **0.034** |
| **Mental illness (partner)** |  |  |  |  |  |  |  |  |  |  |
| No |  |  |  |  | 1 |  | 1 |  | 1 |  |
| Yes |  |  |  |  | 1.10 | 0.759 | 0.91 | 0.795 | 0.76 | 0.487 |
|  |  |  |  |  |  |  |  |  |  |  |
| PERSON |  |  |  |  |  |  |  |  |  |  |
| **Gestational age** |  |  |  |  |  |  |  |  |  |  |
| Normal |  |  |  |  |  |  | 1 |  | 1 |  |
| Preterm |  |  |  |  |  |  | 1.12 | 0.785 | 1.03 | 0.953 |
| **Birthweight** |  |  |  |  |  |  |  |  |  |  |
| Normal |  |  |  |  |  |  | 1 |  | 1 |  |
| Low birthweight |  |  |  |  |  |  | 1.27 | 0.641 | 1.59 | 0.392 |
|  |  |  |  |  |  |  |  |  |  |  |
| PROCESS |  |  |  |  |  |  |  |  |  |  |
| **Child meets relatives, friends, family acquaintances** |  |  |  |  |  |  |  |  |  |  |
| Every day |  |  |  |  |  |  |  |  | 1 |  |
| A few times a week |  |  |  |  |  |  |  |  | 0.79 | 0.358 |
| Once a week or less often |  |  |  |  |  |  |  |  | 1.25 | 0.413 |
| **Shared book-reading** |  |  |  |  |  |  |  |  |  |  |
| Every day |  |  |  |  |  |  |  |  | 1 |  |
| A few times a week |  |  |  |  |  |  |  |  | 0.95 | 0.795 |
| Once a week or less often |  |  |  |  |  |  |  |  | 1.27 | 0.381 |
| **Child sedentary screen time during the week** |  |  |  |  |  |  |  |  |  |  |
| ≤ 1 hour |  |  |  |  |  |  |  |  | 1 |  |
| > 1 hour |  |  |  |  |  |  |  |  | 1.76 | **0.001** |
| **Child sedentary screen time during the weekend** |  |  |  |  |  |  |  |  |  |  |
| ≤ 1 hour |  |  |  |  |  |  |  |  | 1 |  |
| > 1 hour |  |  |  |  |  |  |  |  | 1.25 | 0.274 |
|  |  |  |  |  |  |  |  |  |  |  |
| AU-ROC (with 95% CI) | 0.5702 [0.53042- 0.60991] |  | 0.6180 [0.57971- 0.65623] |  | 0.6220 [0.58363- 0.66042] |  | 0.6254 [0.58373- 0.66715] |  | 0.6684 [0.62620- 0.71050] |  |

Table 7: Results from multiple logistic regressions displaying the odds ratios of having a high ASQ:SE score (above 50) for 3-year-olds girls (complete case analysis).

|  | Context | | | | | | +Person | | +Process | |
| --- | --- | --- | --- | --- | --- | --- | --- | --- | --- | --- |
|  | Family social context  (n= 1340) | p-value | + Parents' lifestyle  (n=1340) | p-value | +Parents' mental health  (n=1340) | p-value | Child characteristics  (n=1168) | p-value | Proximal processes  (n=1083) | p-value |
| CONTEXT |  |  |  |  |  |  |  |  |  |  |
| **Parents' place of birth** |  |  |  |  |  |  |  |  |  |  |
| Both born in Sweden | 1 |  | 1 |  | 1 |  | 1 |  | 1 |  |
| One born in Sweden | 1.44 | 0.170 | 1.44 | 0.180 | 1.48 | 0.145 | 1.26 | 0.465 | 1.06 | 0.881 |
| Both born outside Sweden | 4.18 | **0.002** | 4.42 | **0.001** | 4.70 | **0.001** | 4.81 | **0.002** | 3.40 | **0.034** |
| **Parental education** |  |  |  |  |  |  |  |  |  |  |
| Both more than high-school | 1 |  | 1 |  | 1 |  | 1 |  | 1 |  |
| One more than high-school | 0.68 | 0.094 | 0.69 | 0.109 | 0.68 | 0.102 | 0.60 | 0.059 | 0.62 | 0.091 |
| Neither more than high-school | 1.31 | 0.196 | 1.24 | 0.317 | 1.18 | 0.465 | 1.14 | 0.598 | 1.07 | 0.809 |
| **Family Income (quintiles)** |  |  |  |  |  |  |  |  |  |  |
| 1 (highest quintile) | 1 |  | 1 |  | 1 |  | 1 |  | 1 |  |
| 2 | 1.48 | 0.139 | 1.59 | 0.087 | 1.56 | 0.101 | 1.49 | 0.209 | 1.55 | 0.195 |
| 3 | 0.82 | 0.519 | 0.86 | 0.635 | 0.86 | 0.622 | 0.98 | 0.948 | 0.89 | 0.758 |
| 4 | 0.70 | 0.242 | 0.70 | 0.260 | 0.67 | 0.194 | 0.71 | 0.325 | 0.68 | 0.315 |
| 5 (lowest quintile) | 1.40 | 0.235 | 1.46 | 0.193 | 1.33 | 0.332 | 1.53 | 0.197 | 1.68 | 0.140 |
| **Living arrangement, both parents living together:** |  |  |  |  |  |  |  |  |  |  |
| Yes | 1 |  | 1 |  | 1 |  | 1 |  | 1 |  |
| No | 1.35 | 0.401 | 1.10 | 0.795 | 0.85 | 0.688 | 0.99 | 0.982 | 0.75 | 0.552 |
| **Parents' alcohol habits** |  |  |  |  |  |  |  |  |  |  |
| Neither parent with at-risk use or addiction |  |  | 1 |  | 1 |  | 1 |  | 1 |  |
| One or both parents with at-risk use or addiction |  |  | 1.53 | 0.092 | 1.50 | 0.110 | 1.68 | 0.065 | 1.56 | 0.130 |
| **Parents' smoking habits** |  |  |  |  |  |  |  |  |  |  |
| Neither parent smokes |  |  | 1 |  | 1 |  | 1 |  | 1 |  |
| One or both of the parents smoke |  |  | 1.35 | 0.226 | 1.32 | 0.273 | 1.20 | 0.520 | 1.18 | 0.580 |
| **Parents' drug use** |  |  |  |  |  |  |  |  |  |  |
| Neither ever used drugs |  |  | 1 |  | 1 |  | 1 |  | 1 |  |
| One used drugs once or more |  |  | 1.53 | 0.070 | 1.48 | 0.098 | 1.41 | 0.214 | 1.72 | 0.060 |
| Both used drugs once or more |  |  | 2.03 | **0.035** | 1.71 | 0.120 | 1.98 | 0.057 | 2.65 | **0.011** |
| **Parental stress** |  |  |  |  |  |  |  |  |  |  |
| Neither parent feels stressed a lot |  |  | 1 |  | 1 |  | 1 |  | 1 |  |
| One or both parents feel stressed a lot |  |  | 1.03 | 0.872 | 0.98 | 0.938 | 1.06 | 0.803 | 0.91 | 0.708 |
| **Mental illness (mother)** |  |  |  |  |  |  |  |  |  |  |
| No |  |  |  |  | 1 |  | 1 |  | 1 |  |
| Yes |  |  |  |  | 2.06 | **0.018** | 2.22 | **0.014** | 2.34 | **0.015** |
| **Mental illness (partner)** |  |  |  |  |  |  |  |  |  |  |
| No |  |  |  |  | 1 |  | 1 |  | 1 |  |
| Yes |  |  |  |  | 1.69 | 0.191 | 1.61 | 0.267 | 1.21 | 0.706 |
|  |  |  |  |  |  |  |  |  |  |  |
| PERSON |  |  |  |  |  |  |  |  |  |  |
| **Gestational age** |  |  |  |  |  |  |  |  |  |  |
| Normal |  |  |  |  |  |  | 1 |  | 1 |  |
| Preterm |  |  |  |  |  |  | 2.61 | 0.052 | 2.29 | 0.141 |
| **Birthweight** |  |  |  |  |  |  |  |  |  |  |
| Normal |  |  |  |  |  |  | 1 |  | 1 |  |
| Low birthweight |  |  |  |  |  |  | 0.50 | 0.239 | 0.55 | 0.363 |
|  |  |  |  |  |  |  |  |  |  |  |
| PROCESS |  |  |  |  |  |  |  |  |  |  |
| **Child meets relatives, friends, family acquaintances** |  |  |  |  |  |  |  |  |  |  |
| Every day |  |  |  |  |  |  |  |  | 1 |  |
| A few times a week |  |  |  |  |  |  |  |  | 1.04 | 0.917 |
| Once a week or less often |  |  |  |  |  |  |  |  | 1.19 | 0.662 |
| **Shared book-reading** |  |  |  |  |  |  |  |  |  |  |
| Every day |  |  |  |  |  |  |  |  | 1 |  |
| A few times a week |  |  |  |  |  |  |  |  | 0.86 | 0.582 |
| Once a week or less often |  |  |  |  |  |  |  |  | 2.37 | **0.020** |
| **Child sedentary screen time during the week** |  |  |  |  |  |  |  |  |  |  |
| ≤ 1 hour |  |  |  |  |  |  |  |  | 1 |  |
| > 1 hour |  |  |  |  |  |  |  |  | 1.90 | **0.006** |
| **Child sedentary screen time during the weekend** |  |  |  |  |  |  |  |  |  |  |
| ≤ 1 hour |  |  |  |  |  |  |  |  | 1 |  |
| > 1 hour |  |  |  |  |  |  |  |  | 0.99 | 0.977 |
|  |  |  |  |  |  |  |  |  |  |  |
| AU-ROC (with 95% CI) | 0.6199 [0.57169- 0.66810] |  | 0.6481 [0.60002- 0.69626] |  | 0.6584 [0.61039- 0.70632] |  | 0.6768 [0.62283- 0.73081] |  | 0.7134 [0.65962- 0.76724] |  |
